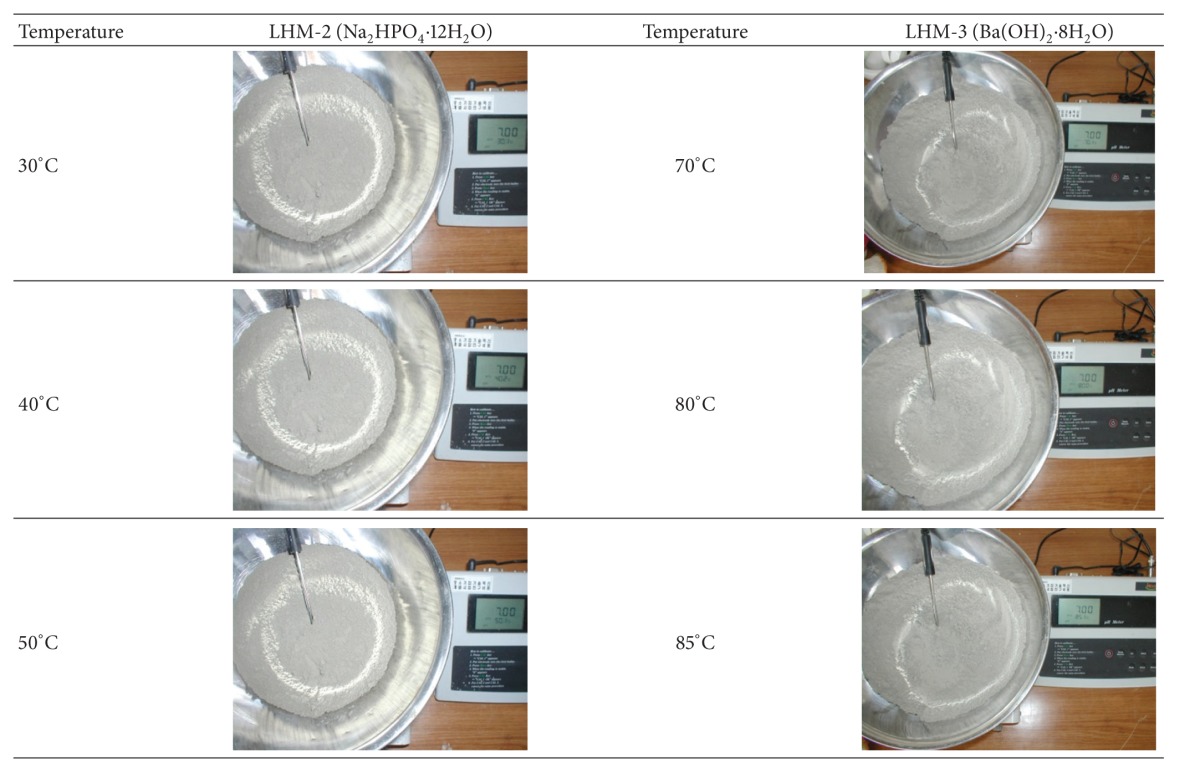# Feasibility of Using Phase Change Materials to Control the Heat of Hydration in Massive Concrete Structures

**DOI:** 10.1155/2014/781393

**Published:** 2014-07-16

**Authors:** Won-Chang Choi, Bae-Soo Khil, Young-Seok Chae, Qi-Bo Liang, Hyun-Do Yun

**Affiliations:** ^1^Department of Architectural Engineering, Gachon University, Gyeonggi-Do 461-701, Republic of Korea; ^2^Tripod Co., Inc., Daejeon 305-335, Republic of Korea; ^3^Department of Architectural Engineering, Woosong University, Daejeon 300-830, Republic of Korea; ^4^Red Butterfly Strontium Industry Co., Ltd, Choungqing 402-368, China; ^5^Department of Architectural Engineering, Chungnam National University, Daejeon 305-764, Republic of Korea

## Abstract

This paper presents experimental results that can be applied to select a possible phase change material (PCM), such as a latent heat material (LHM), to control the hydration heat in mass concrete structures. Five experimental tests (microconduction, simplified adiabatic temperature rise, heat, and compressive strength tests) were conducted to select the most desirable LHM out of seven types of inorganic PCM used in cement mortar and to determine the most suitable mix design. The results of these experimental tests were used to assess the feasibility of using PCM to reduce hydration heat in mass concrete that was examined. The experimental results show that cement mortar containing barium- [Ba(OH)_2_ *·*8H_2_O] based PCM has the lowest amount of total hydration heat of the cement pastes. The barium-based PCM provides good latent heat properties that help to prevent volume change and microcracks caused by thermal stress in mass concrete.

## 1. Introduction

Mass concrete has a high potential to crack due to the temperature difference between the inside and the outside of concrete structures that results from hydration after a large quantity of concrete is placed. As these cracks can be the main cause of weakening the durability and compromising the structural performance of the structure, controlling and decreasing the hydration heat of mass concrete are important.

Various construction methods and new forms of technology to prevent thermal cracking in mass concrete have been studied. Among them, using phase change material (PCM) is a possible way to mitigate the thermal effects in mass concrete. Different types of PCM have been studied and applied to thermal storage materials (e.g., gypsum board, plaster, wall panels, and concrete) as part of building structures over the past years [1–4].

The latent heat of PCM is the heat that is absorbed or radiated as a substance changes its phase from solid to liquid gas (or vice versa) and from liquid to gas (or vice versa). Latent heat and phase transition temperatures differ from one substance to another, which means that each type of latent heat material (LHM) has different latent heat and phase transition temperatures. For this reason, LHMs that are selected according to specific usage can be applied in many areas, including energy-related, chemical, and construction industries. Factors to consider in the selection of appropriate LHMs include usage temperature and the minimum required amount of latent heat.

The high heat absorbance or radiation effect of latent heat can help preserve original energy and maintain a constant temperature. In addition, these characteristics of LHM can stop the rise in temperature of a substance that radiates heat. These characteristics of LHM in mass concrete play a role in mitigating the radical fluctuation of hydration temperature and preventing retarding effects with the use of low heat binder in cement mixtures.

PCMs have been classified into three categories: organic, inorganic, and eutectics [5]. The literature indicates that organic PCMs are preferred over the other two types because of their superior advantages and their manageable disadvantages [2, 6].  Table 1 presents a comparison of the advantages and disadvantages of the three types of PCM [7].

A few countries, such as Korea, Japan, and China, have been working on developing technology that uses encapsulating PCMs as hydration heat reducing admixtures [8–10]. This kind of technology is highly technical in manufacturing capsules and determining the functional sizes of capsules containing PCMs. In addition, there are some doubts about its practical application in mass concrete, despite its good performance in reducing thermal cracking.

Mihashi et al. [11] conducted research that attempts to apply a retarder containing PCM in a paraffin microcapsule to control the hydration heat that is generated in the early phase of hydration. This study hypothesized that the melting of the wax absorbs the heat that is derived from the hydration in a mass concrete specimen. Then, the retarder in the mixture reduces the hydration rate and simultaneously releases heat in the mixture. It is reported in their study that the maximum hydration temperatures under semiadiabatic curing can be lowered significantly in both small cement paste specimens and large concrete specimens.

Microencapsulation of PCM is effectively functional in a fresh concrete mixture, but it adversely affects the mechanical strength of the concrete and causes bleeding during the melting phase of the PCM. Moreover, large-volume containment, or macro-encapsulation, of organic PCM is unsuccessful due to the poor thermal conductivity of organic PCM [12].

This study focuses on evaluating the applicability of seven different types of inorganic PCM, as shown in Table 2, to conditions that are similar to those used for concrete materials. As addressed in Table 1, inorganic PCM usually has a higher density than organic PCM, because inorganic PCM generally is hydrated. Inorganic PCM also is relatively inexpensive and exhibits high latent heat. In addition, the hydrated types of PCM usually are peritectic, which means that they melt incongruently and have a different composition than their original form.

Likewise, PCM is reported [13] in previous studies as being effective in controlling cracks due to the hydration of mass concrete and the resultant autogenous shrinkage. This study attempts to review the characteristics of the hydration heat of binders, focusing on different types of PCM. Based on the results of this study, the most effective PCM will be selected, and an appropriate mixing rate will be proposed for further research.

## 2. Experimental Programs

This research has initiated tests using a selection of PCMs to determine the best candidates to control hydration heat in mass concrete. Seven inorganic PCMs, presented in Table 2, were considered for Series 1 of the testing plan. Microconduction tests of cement pastes, each containing one of the seven PCM types, were conducted using a conduction calorimeter.

After selecting three possible PCMs based on the Series 1 test results, the characteristics of the hydration heat of these three types of PCMs were examined in Series 2 testing by conducting a simple adiabatic temperature rise test of the mortar mixtures. In Series 3, the PCMs selected from the Series 2 test results were divided into high-temperature type and low-temperature type with respect to their phase transition temperatures, as listed in Table 2.

These tests were designed to assess the thermal stability of the particular PCM in the cement mixture. Thermal stability is important in preventing the deformation of materials caused by external heat that is present when the PCMs are stored in a silo or during the premixing stage.

After grouping the materials, heat tests were conducted using the cement paste containing the different types of PCM which is a powder format. As aforementioned, this study attempts to review possible methods that can be used to apply PCM as a binder for concrete mixtures to reduce hydration heat in mass concrete. Considering the productivity in ready-mixed concrete, the PCMs were premixed for practical implementation. Therefore, the LHM-2 and LHM-3 specimens, which showed excellent performance throughout Series 1 and 2 testing, were selected for Series 3 testing. Then, these two types of PCM were mixed with cement and their thermal stability was evaluated.

Series 4 testing was designed to determine the proper mix ratios of the PCMs selected from the previous tests. Seven different mix designs (1.0~3.5% in increments of 0.5%) were considered to examine the characteristics of the hydration heat of the mortar mixes. Finally, Series 5 testing was conducted to test the compressive strength of the mortar mixes with and without the PCMs.  Table 3 provides a summary of the testing plan and the considered parameters.

In Series 1 test, the water-to-binder ratio of the cement paste was 0.4 and the PCMs were replaced with 5% weight of cement. For the mortar in Series 2, 4, and 5 tests, the water-to-binder ratio was 40% and the binder-to-sand ratio was 1 : 2.45.

The cement used in this study is Type 1 ordinary Portland cement (OPC) with a density of 3.15 g/cm^3^ and fineness of 3.21. The absorption rate and density of the saturated surface for fine aggregate are 1.24% and 2.61 g/cm^3^, respectively.

**Figure 1:**
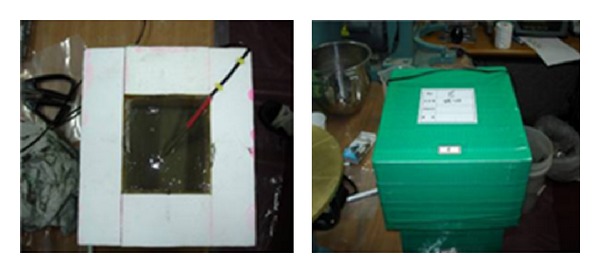
Semiadiabatic temperature rise test setup.

Semiadiabatic temperature tests for each paste were conducted for the specimens tested in Series 2 and 4, as presented in Figure 1. The box used in these tests (100 mm × 100 mm × 100 mm with a thickness of 50 mm) was made with adiabatic materials.

In Series 3, the thermal stability tests utilized a hot plate to monitor the phase change temperature of each binder as the temperature increased. The water-to-binder ratio of the cement paste was 0.35 and the PCMs were replaced with 3.5% weight of cement.

Finally, compressive strength tests of the mortars were conducted in Series 5 in accordance with KS L 5105 [14].

## 3. Results and Discussion

### 3.1. Microconduction of Cement Pastes (Series 1)

**Figure 2:**
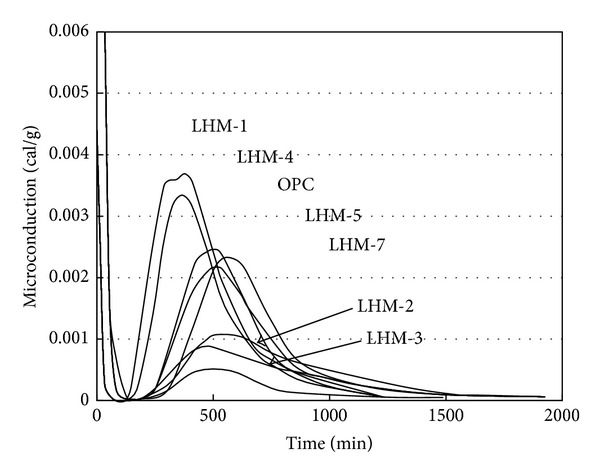
Microconduction measurements for cement paste with each LHM.

Figure 2 shows the microconduction measurements for the cement pastes with the seven types of PCM. The hydration heat of the cement paste with OPC is low, as expected, but it is not low for the LHM-1 and LHM-4 specimens. The latent heat measurements for both the LHM-1 and LHM-4 specimens are relatively higher than for the other PCM specimens. The hydration heat-reducing effect, however, is not significant due to the actions of Na_2_SO_4_ and Na_2_S_2_O_3_ in the chemical compositions of the LHM-1 and LHM-4 specimens, respectively [15].

The microconduction of each type of PCM, except LHM-1 and LHM-4, is shown to decrease as the latent heat increases, although microconduction was not affected considerably by the phase transition temperature of each PCM.

Mixing is required for the PCMs that have high latent heat in order to decrease the hydration heat of the cement paste but not to delay the hydration of the cement paste. Specifically, the LHM-2, LHM-3, and LHM-6 specimens show 50% reduction in microconduction values compared to those of the OPC specimen.

The selected PCMs (LHM-2, LHM-3, and LHM-6) from Series 1 testing were able to maintain an appropriate phase transition temperature to reduce the hydration heat and mitigate abrupt temperature changes in the cement paste.

### 3.2. Adiabatic Temperature Rise Test of Cement Mortars (Series 2)

**Figure 3:**
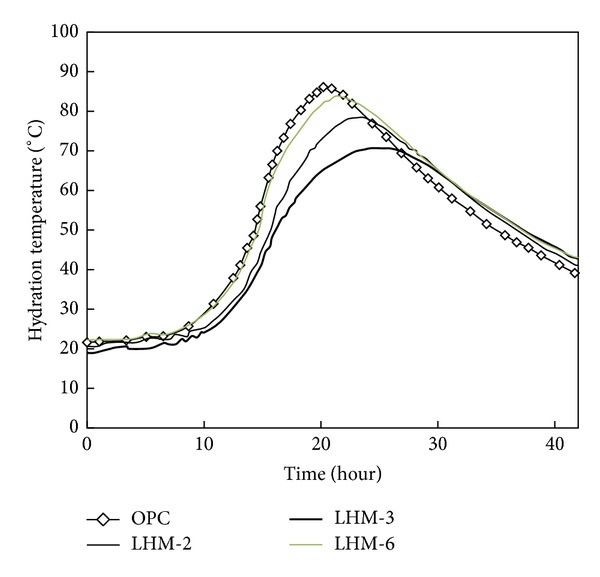
Semiadiabatic temperature rise test.

Figure 3 shows the results of the simple adiabatic temperature rise test of cement mortar containing the three PCMs which were selected based on the Series 1 test results. The maximum hydration temperatures are 85.9°C for the OPC specimen, 78.6°C for LHM-2, 71.1°C for LHM-3, and 84.0°C for LHM-6.

The cement mortar with LHM-3, which has the highest level of latent heat, showed the lowest hydration heat value. The LHM-3 specimen, which was the best in hydration heat-reducing performance, is shown to reduce the hydration temperature by 14.8°C compared to the OPC specimen. The maximum hydration temperature of the cement mortars that contain PCM was lower than that of the OPC specimen regardless of the type of PCM. Moreover, the maximum hydration temperature of the cement mortar that contained PCM was reached slowly compared to that of the OPC specimen regardless of the type of PCM.

### 3.3. Thermal Stability of Cement Pastes (Series 3)

Two PCMs (LHM-2 and LHM-3) selected from the previous test results were used in Series 3 testing.

Table 4 shows the thermal stability test results that include heating the cement paste containing LHM-2 and LHM-3. Deformation of the cement paste with the PCMs was not observed at a temperature of 30°C regardless of the type of PCM. The cement paste with LHM-2, which has a phase transition temperature of 35.0°C, was hardened at about 50°C.

In the case of the LHM-3 specimen, with a phase transition temperature of 78.0°C, no deformation of the cement paste occurred, even at 85.0°C. This result indicates that the application of PCM with a high phase transition temperature is necessary for application in a premixed binder. According to this test's results, the LHM-3 specimen (Ba(OH)_2_ · 8H_2_O) can be used as an alternative material to reduce hydration heat in mass concrete.

### 3.4. Adiabatic Temperature Rise Test with LHM-3 (Series 4)

**Figure 4:**
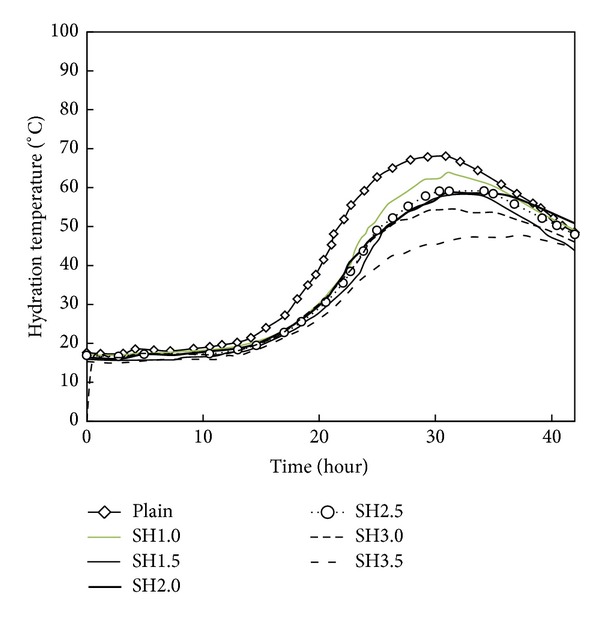
Results of the semiadiabatic temperature rise test according to LHM addition ratio.

Figure 4 shows the results of the semiadiabatic temperature rise tests for the mortars containing various amounts of LHM-3, which is the PCM that showed superior performance in reducing hydration heat in the previous tests.

The hydration heat envelopes of the mortars with respect to the amount of LHM-3 tend to be similar, but the maximum hydration temperature and the time it takes to reach the maximum hydration temperature differ with increasing amounts of LHM-3.

With the addition of 3.0% and 3.5% cement designated as SH3.0 and SH3.5, the hydration temperature decreased by 13.2°C and 20.2°C, respectively. As presented in Figure 4, a considerable reducing effect of hydration heat occurred when the high-temperature type of LHM-3 was added; this effect was more than 3.0% compared to that for the OPC without LHM-3. Moreover, the time it took to reach the maximum hydration temperature was delayed by the addition of LHM-3. In the case of the mortar with 3.5% LHM-3, it took 7.5 hours more to reach the maximum hydration temperature than for the mortar without LHM-3. According to these test results, the hydration heat is reduced and the time needed to reach the maximum hydration temperature is delayed when a high-temperature type of LHM-3 is applied.

In the case of the PCM with a high phase transition temperature, that is, LHM-3, the hydration heat-reducing effect was observed below the phase transition temperature of 78.0°C. This phenomenon resulted in a eutectic effect whereby the substances in the cement and LHM-3 with high phase transition temperatures form a eutectic point at the melting temperature that is higher than each eutectic point. The composite transitions to a liquid state as it melts at a low eutectic temperature.

### 3.5. Compressive Strength Values of Cement Mortars with and without PCM

**Figure 5:**
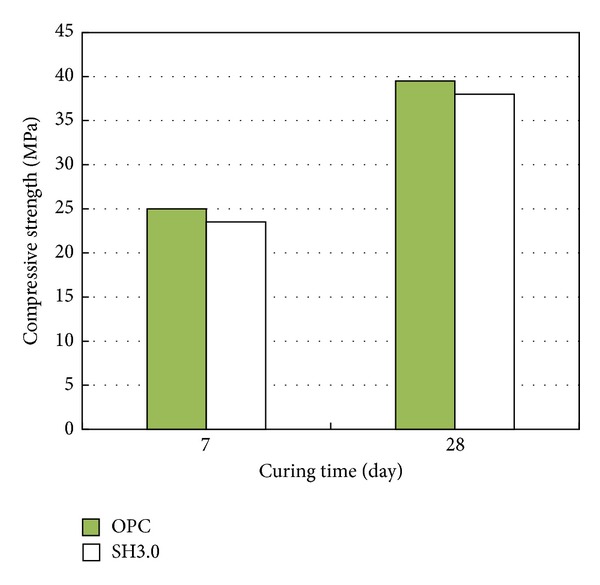
Compressive strength according to 3.0% LHM-3 addition.

Figure 5 shows the compressive strength values at 7 days and 28 days for the control specimen with the OPC and the specimen with the 3.0% LHM-3. The compressive strength values for the specimen with 3.0% LHM-3 are about 4% less than that for the control specimen regardless of the age of the specimen.

The results show that the compressive strength decreases slightly due to the addition of the LHM-3 with a high phase transition temperature. To minimize the adverse effects of the use of LHM-3, further research is needed regarding the mix design and practical implementation in mass concrete.

## 4. Conclusions

In order to develop a method to reduce hydration heat in mass concrete, the characteristics of binders that contain different types of PCM, which have functions in absorbing and releasing heat throughout phase transitions at certain temperatures, have been studied using limited experimental tests.

After selecting seven types of inorganic PCM with a wide range of phase change temperatures, a simple adiabatic temperature rise test was conducted for each PCM selected from Series 1 tests. The PCMs with high latent heat showed superior performance in reducing hydration heat regardless of the phase transition temperature for each PCM type.

The results of the thermal stability tests of the LHM-2 (Na_2_HPO_4_ · 12H_2_O) specimen show that LHM-2 with low phase transition temperatures hardened at 55°C or lower. This result indicates that LHM-2 (Na_2_HPO_4_ · 12H_2_O) is inappropriate for the practical production and admixture of cement-based binder.

The results of the simple adiabatic temperature rise tests show meaningful hydration heat reduction when more than 1.5% LHM-3 is added.

Adding LHM-3 with a high phase transition temperature affects compressive strength values negatively, but the reduction of 4% compressive strength is insignificant and can be mitigated with the development of a mix design that addresses this problem.

Overall, LHM-3 (Ba(OH)_2_ · 8H_2_O) that makes up 3.5% of the cement shows the best performance as a hydration heat-reducing binder in mass concrete. Further research will be conducted regarding its mix design, material properties, and practical implementation.

## Figures and Tables

**Table 1:** Advantages and disadvantages of PCMs [7].

Classification	Advantage	Disadvantage
Organic PCMs	(1) Availability in a large temperature range (2) High heat of fusion (3) No supercooling (4) Chemically stable and recyclable (5) Good compatibility with other materials	(1) Low thermal conductivity (2) Relative large volume change (3) Flammability

Inorganic PCMs	(1) High heat of fusion (2) High thermal conductivity (3) Low volume change (4) Availability in low cost	(1) Supercooling (2) Corrosion

Eutectics	(1) Sharp melting temperature (2) High volumetric thermal storage density	Lack of currently available test data of thermophysical properties

**Table 2:** Properties of PCMs used in this study.

Mark	Substances	Phase transition temperature (°C)	Latent heat (J/g)
LHM-1	Na_2_SO_4_ *·*10H_2_O	32.4	251
LHM-2	Na_2_HPO_4_ *·*12H_2_O	35.0	281
LHM-3	Ba(OH)_2_ *·*8H_2_O	78.0	266
LHM-4	Na_2_S_2_O_3_ *·*5H_2_O	48.0	197
LHM-5	CaBr_2_ *·*6H_2_O	38.2	115
LHM-6	Ca(NO_3_)_2_ *·*4H_2_O	47.0	201
LHM-7	Zn(NO_3_)_2_ *·*6H_2_O	36.0	147

**Table 3:** Summaries of tests.

Series	Tests	Test level	Evaluation item
1	Microconduction for PCMs	7 types of PCM	Microconduction
2	Hydration heat for PCMs	3 types of PCM	Semiadiabatic temperature rise
3	Heat stability for PCMs	2 types of PCM	Heating
4	Characteristics of hydration heat with respect to mix ratio of PCMs	1.0~3.5% (every 0.5%)	Semiadiabatic temperature rise
5	Characteristics of compressive strength with and without PCMs	with/without PCMs	Compressive strength

**Table 4:** Test results of thermal stability in LHM-2 and LHM-3.